# Diospyrobezoar Formation in Patient With Sleeve Gastrectomy

**DOI:** 10.7759/cureus.27561

**Published:** 2022-08-01

**Authors:** Charleston R Powell, Jared S Magee, Ioannis B Papadopoulos

**Affiliations:** 1 Internal Medicine, Weed Army Community Hospital, Fort Irwin, USA; 2 Gastroenterology, Walter Reed National Military Medical Center, Bethesda, USA; 3 Gastroenterology, Madigan Army Medical Center, Tacoma, USA

**Keywords:** sleeve gastrectomy, bariatric surgery, gastric outlet obstruction, endoscopy, bezoar, gastroenterology

## Abstract

The ingestion of Asian persimmons, *Diospyros kaki*, is a known cause of gastric bezoars. Patients with a history of gastric operations are at high risk for formation. Different forms of bariatric surgery have been implicated, but literature for bezoar following a sleeve gastrectomy is scarce. This case report describes the pathogenesis, clinical course, and definitive management of gastric diospyrobezoar following a sleeve gastrectomy. With the rising incidence of bariatric procedures being performed, providers should include bezoar in the differential diagnosis in patients with suspected gastric outlet obstruction and should be aware of treatment options for this patient population.

## Introduction

Gastric bezoars are undigested foreign bodies trapped in the stomach. The classification of a bezoar is determined by the composition and can be formed of plant matter (phytobezoar), hair (trichobezoar), milk products (lactobezoar), or medication consumption (pharmacobezoar). Phytobezoar is the most commonly identified [1].

It is recognized that patients with previous gastric surgeries are predisposed to bezoar formation, most occurring after vagotomy, with incidences ranging from 5 to 12% [2,3]. Furthermore, bezoars have been described in bariatric procedures, such as in patients who have undergone Roux-en-Y gastric bypass (RYGB) or laparoscopic adjustable gastric band (LAGB) [4,5]. However, bezoar formation following the laparoscopic sleeve gastrectomy (LSG) procedure has only been described in several case reports to our knowledge [6-10].

Asian persimmons, *Diospyros kaki*, are edible fruits commonly dried and eaten as a snack. When eaten in excess, the fruit can form a specific phytobezoar called a diospyrobezoar. Here, we report an interesting case of diospyrobezoar formation in a patient with a history of LSG after ingestion of dried persimmons.

This article was previously presented as posters at the ACG 2018 Annual Scientific Meeting & Postgraduate Course in 2018 and the ACP Army/Airforce Annual Meeting in 2018.

## Case presentation

A 40-year-old female with a history of LSG five years prior presented to the emergency department with 10 days of non-bloody, non-bilious emesis. The patient had no prior occurrences of such and did not have a history of diabetes or gastroparesis. Symptoms were most noticeable when eating greasy or fatty foods and were accompanied by nausea, bloating, and epigastric pain. She tolerated liquids, and symptoms were relieved by inducing emesis. Vitals and an abdominal examination were benign. Complete blood count, chemistry and liver function tests were within normal limits at the time of presentation.

**Figure 1:**
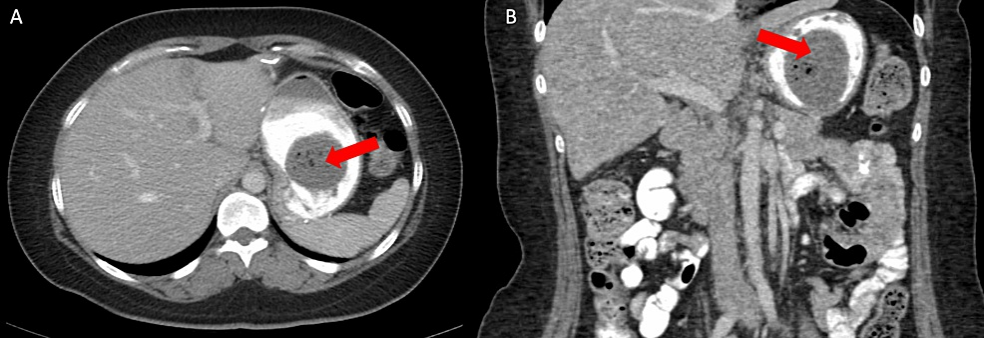
Computed tomography images of gastric bezoar. Computed tomography scan with oral contrast demonstrating gastric bezoar in axial (A) and coronal (B) views (red arrows).

A computed tomography (CT) image of the abdomen with contrast was performed and demonstrated a 4.9 × 4.6 × 5.6 cm heterogeneous gas-containing lesion within the stomach consistent with a gastric bezoar (Figures 1A, 1B).

**Figure 2:**
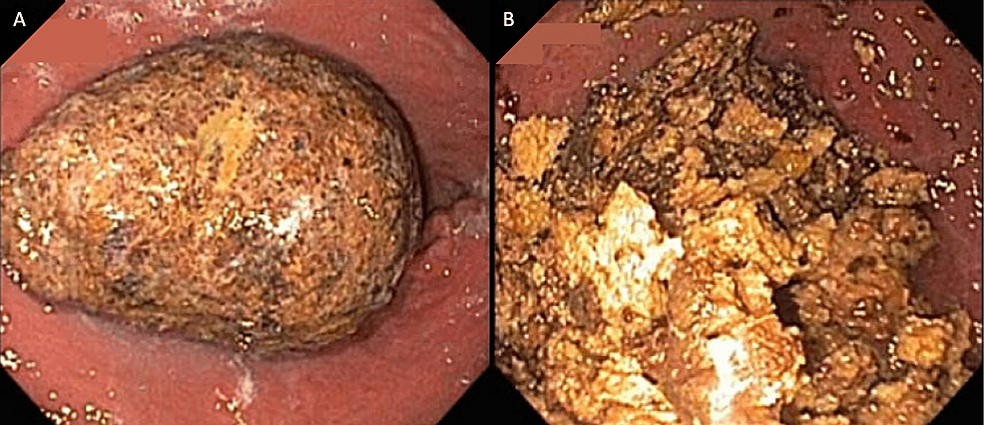
Endoscopic images of gastric bezoar before (A) and after (B) disruption.

The patient was instructed to start drinking cola in an attempt to dissolve the bezoar prior to evaluation in the gastroenterology clinic. Upon further questioning, she endorsed eating a small box of dried persimmons over a one-month timeframe prior to developing symptoms. During esophagogastroduodenoscopy (EGD), there was a large amount of phytobezoar located in the gastric body (Figure 2A). Contents were broken down with snares, forceps, and a bipolar probe (Figure 2B), then removed with a Roth net endoscopically. The gastric outlet was widely patent after the removal of the phytobezoar. Following the procedure, the patient was discharged home with dietary instructions to avoid foods and substances that can predispose to further bezoar development, and there has been no return of symptoms since discharge.

## Discussion

Phytobezoar formation is complex and is thought to be the result of an interplay between mechanical, neuronal, chemical and hormonal changes in a patient, often due to surgery. Consequences of surgery, such as decreased gastric motility and hypoacidity, are considered important factors in phytobezoar formation, in addition to non-surgical factors, such as a patient’s dentition, comorbidities (particularly diabetes and hypothyroidism), and quantity of foods or materials consumed that pre-dispose one to bezoar formation [11]. The formation of disopyrobezoars is unique in that it is secondary to the polymerization of the tannin, cellulose and proteins contained within the persimmon [11]. Animal studies show decreased ghrelin and increased anorectic peptide YY levels in post-RYGB subjects [12].

A review assessing bezoar following bariatric procedures described the time to bezoar formation ranging from two to 84 months post-operatively [13]. Our patient consumed a food known in the literature to form phytobezoars and was in the defined post-operative time frame where these were found to occur in patients with other bariatric procedures. While bezoar following LSG is rarely reported compared to other bariatric procedures, there are several potential explanations postulated. LSG preserves pyloric function and does not require anastomosis that may be prone to stricture. This procedure, along with other contemporary bariatric procedures, does not include vagotomy, which was previously performed in conjunction with a bariatric, ulcer, and anti-reflux procedures [11]. An incisura stricture was posited as a possible explanation for the formation of phytobezoar following LSG in a recently reported case that required re-operation [7]. Another case of phytobezoar occurred after a novel procedure, Nissen-sleeve gastrectomy [10]. Although no stricture was identified in this case, and the LSG was not performed in conjunction with another surgery, this adds to a small body of reported cases of phytobezoar following LSG. The only identified risk factors for the formation of phytobezoar, in this case, were the history of LSG itself and the patient's dietary habits. Prompt acquisition of CT imaging allowed for diagnosis, and early endoscopic therapy allowed for resolution of symptoms and avoidance of complications or need for surgery.

Treatment of phytobezoars can range from conservative therapy with lavage or enzymatic dissolution to endoscopic or surgical therapy. The size and location of the bezoar may dictate which approach will be more successful. For gastric phytobezoars, conservative therapy, such as cola lavage, is the initial intervention of choice, but often endoscopic or surgical removal of the bezoar is needed, as was in this case. Various case reports outline the use of cola, either orally or endoscopically, administered as an initial treatment [4,14-18], but there is no consensus for delivery method, amount or duration of therapy, nor are there randomized studies comparing early endoscopic therapy to conservative therapy as a first approach. This is likely due to the low case volume of phytobezoars. Our patient had no evidence of complicated bezoar (hemorrhage, perforation, mucosal ulceration) on CT or EGD and thus responded well to endoscopic fragmentation and retrieval.

## Conclusions

With the rising incidence of obesity and a concurrent rise in bariatric surgeries performed, clinicians should be attentive to obstructive symptoms from a bezoar. It is important to counsel patients who have undergone these procedures of dietary restrictions, even years after their index operation. Gastric obstruction due to disopyrobezoar following LSG is exceedingly rare but highlights the importance of maintaining a broad differential and obtaining an appropriate dietary history and CT imaging. Endoscopic destruction and retrieval of a disopyrobezoar appears effective following sleeve gastrectomy and should be considered early.
